# Editorial: Methods in T cell biology: 2022

**DOI:** 10.3389/fimmu.2023.1266576

**Published:** 2023-08-08

**Authors:** Enrique Aguado, Matthew E. Call

**Affiliations:** ^1^ Inflammation Program, Institute of Biomedical Research Cadiz (INIBICA), Cádiz, Spain; ^2^ Department of Biomedicine, Biotechnology and Public Health (Immunology), University of Cádiz, Cádiz, Spain; ^3^ Structural Biology Division, Walter and Eliza Hall Institute of Medical Research, Parkville, VIC, Australia; ^4^ Department of Medical Biology, University of Melbourne, Parkville, VIC, Australia

**Keywords:** T cell, TCR (T cell receptor), T cell epitope, method, binding prediction, machine learning

T cells are crucial players of immune responses by their ability to specifically recognize foreign, potentially harmful, antigens. To perform such specific recognition, T cells express at their plasma membrane an antigen receptor called the T cell receptor (TCR) (1–3). The TCR expressed at the plasma membrane in the majority of T cells is composed by a TCR-α and TCR-β heterodimer, which is able to recognize a complex formed by an antigenic peptide coupled to an MHC molecule (pMHC complex). After specific recognition by a pMHC complex the TCR triggers multiple intracellular signals leading to proliferation, target cell killing, cytokine secretion, and differentiation into effector T cells. Although our understanding of the molecular mechanisms governing activation of T cells has dramatically grown in recent years, there is still much to learn.

This Research Topic contains 5 articles covering different aspects in the field of experimental techniques and methods used to investigate fundamental questions in T Cell Biology. Indeed, new technologies have recently appeared that have not only given us a much better understanding of these cells, but have also allowed researchers to genetically modify T cells for the treatment of malignant tumors. The study of TCRs is of crucial importance for fine-tuning immunotherapy of cancer and human infectious diseases. One of the articles in the present topic addresses a review and comparison of different deep learning methods to investigate TCR-peptide/-pMHC binding prediction, as a means to generate tools for the treatment of tumors or infectious diseases (Grazioli et al.). In their work, Grazioli et al. have tested the reliable performance of state-of-the-art deep learning methods in predicting TCR/pMHC peptide binding for “unseen” peptides, i.e., sequences that were not present in the training set. For this purpose, they have integrated TCR-peptide/pMHC samples from different databases into a single database (which they have named TChard), and with it they have performed experiments with two state-of-the-art deep learning models for TCR-peptide/pMHC interaction prediction. Their results show that those deep learning methods fail to generalize to unseen peptides, unveiling the need of more robust TCR-peptide/-pMHC interaction prediction, machine learning models. Also of interest is the work presented by Hong et al. in which a TCR cloning system not needing single cell technology is described. In their work they use human cytomegalovirus (CMV)-derived peptides, as it is one of the causes of acute graft-versus-host disease (GVHD) after hematopoietic stem cell transplantation, and adoptive transfer of CMV-specific T cells has been shown to restore immune functions against CMV infection (4). They had previously shown that specific HLA class I allotypes are preferentially used in immune responses to pp65 mediated by CD8 + T cells (5). Here the authors establish a rapid method for the identification of functional TCRs from CMV pp65 antigen-specific T cells restricted by particular HLA allotypes, and a reverse TCR cloning method for amplifying only specific TCRs from the bulk TCR cDNA pool.

Schollhorn et al. describe a method to identify by flow cytometry activated T cells, with great sensitivity. They assess activation of CD4+ and CD8+ T cells using a combination of β2-integrins, CD137 and CD154. The interest of this method lies in the fact that the analysis of the expression of β-2 integrins correlates well with cytokine production and preserves cell viability. This approach not only allows detection and quantification of activated T cells in a shorter time than other activation markers (e.g. CD25 or OX40), but also has a lower background staining than CD69. The authors show in their report the potential utility of their method by assessing specific T cell responses to the SARS-CoV-2 spike protein in individuals following vaccination with COVID-19.

With regard to T cell activation, it is well established that three-dimensional organization of the genome, i.e., the organization of chromatin compaction, is intimately associated with lymphocyte development and activation (6). Dynamic allelic interactions and nuclear locations seem to be especially relevant for the regulation of immune responses. In this context, Salataj et al. have published in this Research Topic a detailed protocol to simultaneously detect nascent RNA transcripts (3D RNA FISH), their genomic loci (3D DNA FISH) and/or their chromosome territories (CT paint DNA FISH), in combination with the antibody-based detection of several nuclear factors. In their article, the authors describe the application and efficacy of this protocol in various subtypes of T cells, B cells, macrophages and other cell types. It is of interest to note that this is a very detailed protocol, with a section in which the authors propose several optimizations for potential problems that may arise in the implementation of the method.

On the other hand, after specific TCR stimulation, T lymphocytes are metabolically activated, and the mitochondrial network is of special relevance for proper immune responses. In the last article in this Research Topic, Gómez-Morón et al. describe the function of EB1 (a protein that regulates tubulin polymerization and previously identified as a regulator of intracellular transport of CD3-enriched vesicles) in Jurkat and primary T cells. In their work the authors show that the decrease in EB1 expression produces deficient intracellular organization and metabolic strength after T cell activation T cells, suggesting a link between the cytoskeleton and metabolism in response to TCR stimulation, which leads to increased AICD.

## Author contributions

EA: Writing – original draft, Writing – review & editing. MC: Writing – review & editing
